# P296: Epidemiological profile and management of accidental blood exposure, Bamako

**DOI:** 10.1186/2047-2994-2-S1-P296

**Published:** 2013-06-20

**Authors:** A Traoré, M Dembélé, DS Ouologuem, DK Minta, AS Kaya, T Cissé, HA Traoré

**Affiliations:** 1 Service of Infectious Diseases, University Hospital of Point G , Bamako, Mali; 2 Department of Internal Medicine, University Hospital of Point G , Bamako, Mali

## Objectives

The aims of this study were to describe the epidemiological profile, circumstances of occurrence and post-exposure practices.

## Methods

We conducted a retrospective study on records of people who were victims of accidental exposure to blood (AES) treated in the Department of Infectious Diseases, in the University Hospital Teaching of Point G, Bamako-Mali over 8 years.

## Results

We collected 40 AES with a male predominance (sex- ratio = 2). The median age was 32 years [19-54 years]. Our study population was composed of students (42.5%), physicians (22.5%), nurses (15%) and surface technicians (sanitizers) (7.5%). The AES had occurred mainly in hospitals (57.5%) and 20% in home care. The surgery department was more concerned (45%). The main circumstances of occurrence were the needle stick (42.5%); puncture by scalpel (22.5%); recapping (10%) and the projection on the mucosa (10%). The majority had done the washing with water / soap (92.5%) of which 25% immediately. Eight people were reported after 48 hours. HIV status of the source patient was known in 47.5% of which 25% were positive. A lady had a positive HIV serology after accidental blood exposure (seroconversion) At the time of AES, serology for hepatitis B and C were not known in the majority of patients and victims sources. Thirty have benefited from ARV prophylaxis.

## Conclusion

Difficulties encountered in the declaration and management of AES require training of health personnel of University Hospital Teaching of Point G.

## Competing interests

None declared

